# Factors Affecting Yeast Digestibility and Immunostimulation in Aquatic Animals

**DOI:** 10.3390/ani14192851

**Published:** 2024-10-03

**Authors:** Sadia Sultana, Janka Biró, Balázs Kucska, Csaba Hancz

**Affiliations:** 1 Kaposvár Campus, Hungarian University of Agriculture and Life Sciences, Guba S. 40., 7400 Kaposvár, Hungary; sadiazoology@nstu.edu.bd (S.S.);; 2 Research Center for Fisheries and Aquaculture, Hungarian University of Agriculture and Life Sciences, Anna-liget u. 35, 5540 Szarvas, Hungary

**Keywords:** yeast, fish, growth performance, aquafeeds, digestibility, immunostimulation

## Abstract

**Simple Summary:**

This paper provides an overview of the key issues concerning the use of yeast products as an alternative protein source and feed additives to develop a more sustainable and environmentally friendly aquaculture. The use of yeast is presented in detail, with a particular focus on its use as a primary protein source and as a supplement (such as probiotics and immune stimulants). Additionally, the digestibility of yeasts and their use in live aquafeed culture are discussed in detail.

**Abstract:**

The aquafeed industry increasingly relies on using sustainable and appropriate protein sources to ensure the long-term sustainability and financial viability of intensive aquaculture. Yeast has emerged as a viable substitute protein source in the aquaculture sector due to its potential as a nutritional supplement. A substantial body of evidence exists to suggest that yeast has the potential to act as an effective immune-stimulating agent for a range of aquaculture fish species. Furthermore, the incorporation of yeast supplements and feed additives has the potential to bolster disease prevention, development, and production within the aquaculture sector. Except for methionine, lysine, arginine, and phenylalanine, which are typically the limiting essential amino acids in various fish species, the various yeast species exhibit amino acid profiles that are advantageous when compared to fishmeal. The present review considers the potential nutritional suitability of several yeast species for fish, with particular attention to the various applications of yeast in aquaculture nutrition. The findings of this study indicate that the inclusion of yeast in the diet resulted in the most favorable outcomes, with improvements observed in the overall health, growth performance, and nutritional condition of the fish. Digestibility, a key factor in sustainable feed development, is discussed in special detail. Additionally, this review addresses the utilization of yeast as an immunostimulating agent for fish and its digestion in fish. Furthermore, the research emphasizes the necessity of large-scale production of yeast as a substitute for fishmeal in aquaculture.

## 1. Introduction

Aquaculture is an essential component of the global animal production system to meet the long-term protein needs of the world’s population [1]. In recent years, the global aquaculture industry has seen an increase in the importance of using cutting-edge nutritional techniques to improve fish health and disease resistance [2]. Aquaculture must continue to expand sustainably to meet the future demand for animal protein and support the continued growth of the global population [3]. The sector has encountered obstacles to its rapid growth, including diseases and restricted access to high-quality protein sources for fish feed. In aquatic species, protein represents the primary dry matter component of growth [4]. Aquafeeds are formulated to include all the essential nutrients that fish require to maintain optimal health. Fishmeal (FM) and fish oil (FO), which are derived primarily from wild-caught small pelagics like anchovies, sardines, and menhaden, have historically been a significant component of aquaculture diets. According to IFFO, approximately 69% of FM and 75% of FO are utilized in aquaculture production [5]. In particular, the proportions of fishmeal and fish oil have significantly decreased and have been substituted with components derived from plants. Despite changes in feed composition to reduce marine ingredient dependency, the sustainable development of aquaculture faces threats from stagnant fishmeal and fish oil supply [6]. Furthermore, fishmeal and plant-based meals are currently utilized as primary sources of protein. Consequently, sustainability concerns have necessitated the identification of novel sustainable components for aquafeeds [7]. Compared to animal-sourced feedstuffs, plant-sourced feedstuffs frequently exhibit reduced dry matter contents of amino acids (AAs) and crude protein (CP) [4,8]. The reduction in fish stocks and the elevated cost of fishmeal have created significant challenges for the aquaculture industry in meeting its consumption requirements. It is therefore a global priority to identify alternative protein sources that can be used to replace fishmeal in aquafeeds [9]. Over the next ten years, it is predicted that fisheries’ trimmings and byproducts will provide increasing fishmeal and oil production [10]. However, they will likely not be sufficient to meet anticipated demand by 2050 [11], which may also have an impact on the nutritional value of farmed fish. In response to these growing concerns, large-scale research has recently been conducted to identify acceptable fishmeal substitutes that can be included in the diets of a variety of aquatic animal species. To address these challenges, novel aquafeed materials have been developed, including macroalgae, insects, genetically modified crops, and single-cell proteins derived from microalgae, bacteria, or yeasts [12]. Furthermore, single-cell protein sources, particularly yeast protein products, may serve as a supplementary solution to offset the adverse effects of plant proteins [13]. 

Recent studies have demonstrated the potential of yeast as a viable alternative protein source in aquaculture, offering a promising solution to replace fishmeal [7,14]. Research findings have shown that yeast can be a sustainable and high-quality protein source in fish diets, with favorable amino acid composition and health benefits [15]. The latest advances in yeast research have focused on its potential as a functional ingredient and nutritional supplement that can enhance fish immune systems and gut health [3]. Recent advances in the study of yeast have focused on its potential as a functional component and dietary supplement that strengthens the immune systems and gut health of fish. In aquaculture, *Saccharomyces cerevisiae* and its byproducts are the most often utilized functional feed additives [16,17]. Yeast supplements have improved immunity and enhanced aquaculture water quality, leading to better productivity and disease protection [18]. Dietary yeast extracts have also been found to promote the growth of beneficial bacteria and inhibit some pathogenic species in fish [19]. For instance, supplementing the diet of largemouth bass (*Micropterus salmoides*) with brewer’s yeast hydrolysate inhibited bacterial members of the genus *Mycoplasma* and significantly increased *Cetobacterium* in their intestines [19]. Furthermore, yeast-derived cell wall fractions have been shown to have immunological and health benefits for fish [15]. Several scientific reviews have explained the health benefits of these cell wall components in different species over the years, but little is known about the function of yeast as a macro-ingredient in fish feeds [20]. However, supplementing with *S. cerevisiae* as a probiotic has improved the growth, immunity, and disease resistance of various fish species [21]. Probiotic fermentation can help remove toxicity from dietary ingredients used as alternative protein sources to replace fishmeal [22]. In addition, yeasts have been used to develop artemia and rotifers or to ferment feedstock following artificial or natural colonization in the gastrointestinal tracts of the hosts [18,23]. Other findings underscore the value and sustainability of yeast products as a nutritional source in aquaculture. Their probiotic effects also enhance water quality [24]. 

This review aims to present an overview of current knowledge regarding the use of yeasts as a primary source of protein, supplements, and probiotics in aquaculture systems. In particular, it will emphasize the effects of these organisms on the growth performance, digestibility, physiology, and immune system of aquatic species. 

## 2. Yeast Cell Wall Components and Their Nutritional Properties

Yeast, a single-celled eukaryote with membranous organelles such as mitochondria, nucleus, and endomembrane system, is a protein source in aquafeeds for shrimp and marine fish. Many yeast species are frequently utilized, such as Saccharomyces cerevisiae*, Kluyveromyces*, *Torulaspora*, *Saccharomyces*, and *Torulopsis* [14]. The yeast cell wall provides structural support and is made up of 10–15% proteins and 85–90% polysaccharides [25].

The mucosal immune responses of rainbow trout were evaluated when supplementing an experimental formulated feed with multi-strain yeast fraction product of *Saccharomyces cerevisiae* and *Cyberlindnera jardinii* and found that the extracts enhanced and strengthened the ability of innate immune cells of mucosal-associated lymphoid tissues of fish [26]. 

Another study aimed to characterize the biochemical and molecular properties of four proprietary yeast cell wall extracts from *S. cerevisiae* about their potential effect on the intestinal immune responses when given orally to zebrafish (*Danio rerio*), concluding that these characteristics give a chance to correctly evaluate the immune potential of the tested products. Additionally, this study offered novel perspectives on the development of specific fractions derived from *S. cerevisiae* for use in precision animal feeds [27]. 

The estimated polysaccharide content of the S. cerevisiae cell wall is 30–60% b-1, 3-glucan and 1, 6-glucan, 25–30% mannan, and 5–10% chitin [25]. Table 1 shows that yeast *S. cerevisiae* contains approximately 32–62% protein [28]. On average, 93–97% of dry yeast is composed of dry matter, which includes 40–60% crude nitrogen and poultry byproducts protein, 5–9% lipids, and 35–45% carbohydrates [28,29].

Additionally, it is also stated that yeast species such as *S. cerevisiae* have unsaturated fatty acids, and linoleic and alpha-linolenic acids, which can also be formed by fatty acid desaturase [28]. Low-value and non-food cellulosic materials are fermented to produce dry yeast products, via the pre-treatment of biomass by the extraction of lignin; hydrolysis to release cellulose and hemicellulose; enzymatic hydrolysis to release hemicellulose and cellulose into C5 and C6 sugars; fermentation of sugars, ammonia, phosphorus, and other nutrients; and downstream processing into dry yeast products for use as a source of protein in fish feed [15,18].

It can be stated in general that the characteristics of yeast products make them ideal feed ingredients, serving as valuable protein sources. Additionally, their role as pre- and provitamins is of significant importance.

## 3. Use of Yeast in Fish Feeding

As mentioned above, fishmeal is a highly digestible feed ingredient for animals and a great source of high-quality protein, fatty acids, and readily available minerals and vitamins [31]. Finding local raw resources is necessary since the high cost of imported raw materials drives up the price of locally produced feed [32]. Fishmeal inclusion levels in aquafeed must also be lowered to save costs and save the environment [12]. Current research on fish nutrition and aquafeed production primarily focuses on assessing the potential of non-conventional protein sources. These sources include plant-derived proteins, processed animal byproducts, and single-cell proteins [33,34].

As a high-nutritional eukaryotic organism, yeast is an appropriate candidate for use as animal feed [35]. One of the most significant qualities of yeast as a potential feed ingredient is its capacity to transform industrial biomass, forestry waste, and less valuable, inedible food byproducts into high-quality food or feed ingredients with little to no dependence on arable land and water. Furthermore, the use of yeast as a feed ingredient results in a net reduction in climate change [36,37].

Furthermore, the utilization of yeast as a fish feed represents a potentially cost-effective and environmentally sustainable approach within the domain of aquaculture biotechnology, which could diminish reliance on fishmeal [38]. Fishmeal or plant proteins can be substituted with the affordable yeast *S. cerevisiae*, which has been shown to have no detrimental effects on the growth and nutritional performance of rainbow trout [39], Nile tilapia [40], and Arctic charr [41]. A study on alternate sources of protein also reveals that *S. cerevisiae* is an effective natural resource of protein in the replacement of fishmeal in tilapia feed [42]. Similarly, another study found that the 15% inclusion of yeast had increased the growth performance without reducing the quality of the end product [43]. The productivity, immunity, and overall health of Nile tilapia (*Oreochromis niloticus*) have all been improved by the use of fermented yeast products [17]. Recent advances in yeast research are more focused on their potential as nutritional supplements and functional properties with beneficial effects on the immune responses and gut health in fish [3].

The use of yeast as an aquatic feed ingredient is undoubtedly aligned with the developmental objectives of sustainability, resulting in both economic and environmental benefits. The extensive literature overview encompasses a vast array of products, doses applied, species fed on, and effects evaluated. To facilitate a summary, a division was applied as seen below. However, it must be acknowledged that this division may appear somewhat forced at times, given that multiple effects were measured and evaluated in the majority of the studies discussed here.

## 4. Use of Yeast as the Primary Protein Source in Aquafeed

Yeast cells have high protein content (approximately 40–55%) and other bioactive compounds that are essential for fish development and growth performance [7,39]. A multitude of research investigations have demonstrated the beneficial impacts of incorporating whole yeast and/or its byproducts into the diet on Nile tilapia growth performance, hematological indices, antioxidant defense systems, immunological response, and disease resistance [17,44]. Research suggests that a diet enriched with *Saccharomyces cerevisiae* (SC) can improve the growth performance, intestinal morphology, redox homeostasis, and immune response of *S. aurata*. The most effective concentration was found to be 4 g/kg [45]. Candida species are commonly used in rainbow trout diets, and up to 40% of fishmeal can be effectively substituted without any reduction in production or efficiency [14]. In a study, it was revealed that by increasing the feed conversion ratio and body nitrogen retention, a 15% protein-rich yeast fraction inclusion could enhance the growth performance of trout fed on plant-based diets [13].

Brewer’s yeast, mainly the *Saccharomyces cerevisiae* strain, has been used as nitrogen-rich ingredients in aquaculture feeds from the beginning of the 1990s [46]. A study was conducted to assess the suitability of brewer’s spent yeast (BSY) in aquafeed for *Labeo rohita* growth, hemato-biochemical parameters, and enzyme activities, and fishmeal was gradually replaced at an increased level [47]. The study found that replacing 30% of fishmeal with brewer’s wasted yeast considerably improves growth and immunity in *L. rohita*. In addition, another study revealed that replacing 45% of fishmeal with brewer’s yeast can improve the growth performance and immune response of the Thai Panga (*Pangasianodon hypophthalmus × Pangasius bocourti*) [48]. In addition, it was suggested that dried yeast could replace up to 30 and 50% of the protein provided by FM/SBM (50/50%), respectively, without affecting the performance of red drum (*Sciaenops ocellatus)* [49]. In the research, it was concluded that the growth, immunity, and disease resistance of channel catfish were enhanced by replacing fish feed with yeast culture, and the intestinal microbiota of the fish, which was induced by the yeast culture, was a key factor in these outcomes [50]. Furthermore, dietary fermented yeast could be used as a strategic option to maintain tilapia production [17]. Moreover, yeast can convert low-value biomass leftovers from agriculture and forests into high-value feed components, making it a sustainable and environmentally friendly element [37]. Consequently, it follows that fishmeal can be substituted with single-cell yeast protein at certain levels to reduce the aquafeed cost and preserve the sustainable output of aquaculture for the mentioned aquatic species. The effects of yeast products on different fish species are summarized in Table 2.

The results of the experiments demonstrate that the majority of animals exhibited good growth performance when provided with yeast products as a primary protein source. In instances where this trait did not outperform the control, other beneficial effects were observed. However, given the significant diversity in cultured species and age groups, as well as the range of products utilized, a continuous optimization of feed formulations is essential.

## 5. Use of Yeast as Supplement in Aquafeed

Yeast supplements and feed additives improve fish health and production, leading to increased growth in the aquaculture industry [18]. Yeast-based diets are high in protein, lipids, attractants, and other nutrients. *Candida* sp., *Kluveromyces* sp., and *Phaffia* sp. are three yeast species commonly utilized as fishmeal alternatives. Several studies have studied the effects of partial and complete substitution of fishmeal with brewer’s yeast on the growth, body composition, feed consumption, and digestibility of juvenile tilapia [23]. Another study revealed that yeast supplement is a promising growth promoter and might be an alternative method to antibiotics for disease prevention of *Mystus cavasius*) [23]. Selenium is a crucial dietary mineral for immune system function, cell growth regulation, and stress response (Se). It has been observed to improve growth performance, feed utilization, and immunocompetence in a variety of fish species, including Wuchang bream (*Megalobrama amblycephala*) [68], hybrid striped bass (*Morone chrysops* × *Morone saxatilis*) [69], and rainbow trout (*Oncorhynchus mykiss*) [70]. Some studies reported that the dietary fishmeal inclusion level could be reduced to 14% with SPC (Soy Protein Concentrate) [71] or a blend of soybean meal and poultry byproduct meal [72] serving as the fishmeal substitute. In a recent study, it was proven that the dietary fishmeal inclusion level for golden pompano could be reduced to 240 g.kg^−1^ with soy protein as a fishmeal alternative and 1 g kg^−1^ of Se-yeast supplementation. The loss in growth in golden pompano fed a high soy protein-based diet could be due to selenium (Se) deficiency rather than low feed intake and utilization [73]. However, there hasn’t been enough research conducted on how dietary yeast affects the replacement of meals.

A study revealed that the inclusion of yeast in fermented poultry byproduct meal (pbm) in the diet of common carp (*Cyprinus carpio*) at 15–20% level increased digestive enzyme activities, immune function, and the growth of the fish [74]. In contrast, it has been shown that common carps can utilize a high percentage of their dietary protein requirement from the yeasts *Candida tropicalis*, *Candida utilis*, and *Candida lipolytica* with better results than those obtained with soybean or meat and bone meal. Furthermore, it was revealed that various levels of dried yeast in fish feed results in significant impact on body weight of fish as indicated by 20% greater growth by the dietary yeast inclusion in the replacement of fishmeal up to 40% [43]. Likewise, many yeast species have a high impact on the growth performance, feed efficiency, and body composition of different aquaculture species, as shown in Table 3. Therefore, to lower the cost of aquafeed for the sustainable production of aquaculture for these species, it can be deduced that fishmeal should subsequently be substituted at these levels with single-cell yeast protein [75].

The findings of the literature review indicate that the evaluated yeast products effectively functioned as prebiotics and probiotics, and the animals fed them exhibited favorable growth, feed conversion, and survival rates. However, due to the ongoing advancement in methodology for measuring and evaluating the impact of immunostimulation and microbiome diversity enhancement, direct comparisons of the results are challenging.

### 5.1. Use of Yeast as Probiotics in Aquafeed

Aquaculture is expanding worldwide, yet adverse circumstances of fish culture can cause disease and financial loss. Probiotics are being promoted as a safe and sustainable alternative to traditional aquaculture [90]. In addition, probiotics are considered as live or dead microorganisms, or microalgae or yeast that give health advantages when supplied in sufficient doses [91]. Additionally, probiotics can improve fish health by enhancing feed conversion and digestion, reducing stress sensitivity, and raising overall health [92]. Additionally, probiotics stimulate the secretion of intestinal mucus, promote the growth of microvilli, and create barriers against invading pathogens [21]. The scientific community is searching for alternatives to lessen the misuse of antibiotics because they impair fish immunity, harm the aquatic ecosystem, and are present in fish tissue, which can be harmful to consumers’ health [93]. Additives with prebiotic properties from yeast byproducts are used in animal diets to promote growth, improve digestion, enhance microorganism activity, and enhance immune system functionality [94]. Yeasts are resistant to antibacterial medications, making them useful as probiotic supplements during antibiotic treatment. *Saccharomyces cerevisiae* and *S. cerevisiae var. boulardii* (or *S. boulardii*) are widely explored as probiotic yeasts from various sources [95,96,97]. The addition of supplements containing yeast probiotics to fish feed has been shown by several researchers to enhance growth rate [18,40,42], feed digestion [18,23,39], stress tolerance [40], the immune system [18,23], and disease control [40,42,98]. Studies have shown the individual and combinational effects of probiotics (*B. subtilis*, *B. licheniformis and S. cerevisiae*) on the growth, body composition, digestive enzymes, and intestinal morphology of *A. persicus* fingerling and also suggested that the bacterial probiotics might be more effective than the yeast [99]. Probiotics are an environmentally benign way to boost fish health and growth in tilapia farming, as they can replace antibiotics. The study suggests that the use of probiotics, prebiotics, and synbiotics in tilapia aquaculture can significantly enhance growth and immunity, requiring an optimal dosage and administration time [100]. In addition, probiotics and prebiotics have emerged as ecologically benign alternatives to antibiotic treatments in shrimp farming, as a result of the increased interest in investigating alternative approaches [101]. In addition, research into whiteleg shrimp *Penaeus vannamei* has demonstrated that the use of mixed probiotic cultures improves viability, feed conversion, and the final yield of farmed shrimp [102].

In a study, the effect of probiotics was analyzed on survival, welfare, growth indices, and blood composition in Siberian sturgeon (*Acipenser baerii*) raised in a recirculating system. The results revealed that adding yeasts as probiotics to the meals of *Acipenser baerii* fed in a circulating system increased the fish’s growth, survival, and well-being [92]. In addition, it was suggested that probiotic-rich diets can enhance the immunological response and growth of Siberian sturgeons [103]. It has been demonstrated that probiotics positively impact the growth, survival, and health of aquatic animals. Probiotics have been included in aquaculture methods to address the requirement for greater disease resistance, enhanced aquatic organism growth, and feed efficiency [104,105]. The individual and combined effects of probiotics in Persian sturgeon (*Acipenser persicus*) were assessed by measuring growth performance, proximate body composition, digestive enzymes, and intestinal morphology. The findings contributed to a growing body of literature about the individual and combined effects of probiotics with *S. cerevisiae* on growth, body composition, digestive enzymes, and intestinal architecture of *Acipenser persicus* fingerling [99]. Furthermore, commercial probiotic products are currently being made from several species of yeast, such as *S. cerevisiae*, as indicated in Table 2. It can be concluded that the use of yeasts as probiotics is expanding, and it is advantageous to consider these yeasts as a potential substitute for improving development, digestibility, survival rate, feed efficiency, and capacity to fortify the immune systems of fish larvae and juveniles. A summary of the effects of yeast products as probiotics can be found in Table 4.

### 5.2. Use of Yeast as Immune-Stimulant for Fish 

Yeast meal used in aquaculture has been proven to enhance growth, modulate gut microbiota, and boost fish immune systems. Yeast has emerged as a sustainable innovative element in aquafeed due to its potential benefits for immunostimulation and nutrition of several species in aquaculture [18]. To varied degrees of success, numerous studies have been conducted to determine the effects of these immunostimulants on fish development, intestinal morphology, and overall health. In addition to its probiotic effects, *Saccharomyces cerevisiae* can be utilized as an immunostimulant for fish raised in captivity that can benefit from *Saccharomyces cerevisiae’s* ability to promote growth and disease resistance [108]. Research on the potential of yeast as a possible fish feed ingredient is scanty, with a lot of focus on *S. cerevisiae* [3]. Few studies assess the health benefits of using yeast as a source of protein, even though positive health effects have been reported when using entire *S. cerevisiae* yeast or yeast derivatives. Low levels of *S. cerevisiae* yeast have shown to enhance growth performance, immune responses, and/or protection against bacterial infection and increase disease resistance in several fish species, such as salmonids [109], common carp [74], Japanese seabass (*Lateolabrax japonicas*) [60], and Nile tilapia [110]. Yeasts such as *C. utilis* have also been shown to modulate immune responses in rainbow trout, [111] and *K. marxi-anus* has shown probiotic properties in an in vitro colonic model system [112]. Many researchers are interested in yeast cell wall derivatives because of their immunostimulatory qualities and, in part, since the use of chemotherapy in animal feed is prohibited. The majority of recent developments in the study of yeast cell walls, however, have focused more on polysaccharides devoid of the lipid moiety because it has also been noted that these molecules interact with immune system cells and chemicals related to humoral immunity [113]. Additionally, it has been reported that immunostimulatory polysaccharides promote the differentiation, proliferation, and improved function of macrophages by generating reactive oxygen species (ROS) [113].

**Figure 1:**
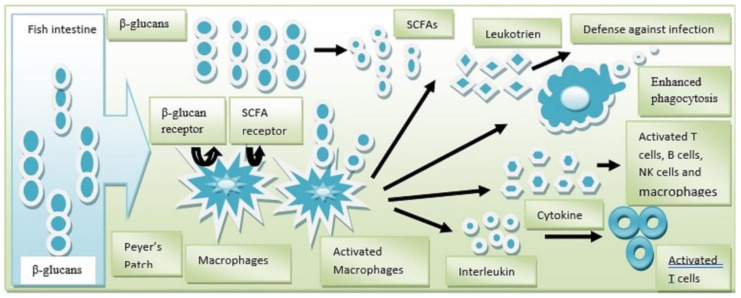
Signaling pathway for yeast β-glucans in the direction of fish immunization. Figure based on Mahdy et al. Source: [18].

Yeasts are a good source of β-glucan which is well known for its role in immunostimulation in fishes and other vertebrates [114]. β-glucan from yeast is crucial for fish development as it boosts the immune system and protects against infections in various ways. In addition, β-glucans facilitate several biological processes such as wound healing [115], stress tolerance [116], and antioxidant [117] and antibacterial processes [118]. The most commonly utilized β-glucans for studying mechanisms of action are those of commercial origin, with recommended doses from manufacturers. Nonetheless, their immunological activity is ineffective in other fish species, and increasing the dose may have negative consequences, including immune suppression [119]. β-glucans are considered as one kind of pathogen-associated molecular pattern (PAMP) [120]. They therefore cause fish to develop a signaling route, and the signaling pathway has been explained and exemplified in Figure 1 using the most recent fish β-glucan research. In addition, they are applied to farm animals to increase feed conversion ratios (FCR), replace antibiotics in the guts of fish, eradicate opportunistic bacteria, enhance intestinal morphology and function, and enhance both innate and acquired immune functions [121], a concept originated from the discovery that certain sugars, like mannose, could be used as inhibitors of pathogen adhesion to intestinal cells [20].

Several studies have assessed the effects of probiotics on the immune status and gut microbiota of European sea bass, (*Dicentrarchus labrax* L., 1758). The investigated aspects range from antioxidant and digestive enzyme activities, growth performances, gut histology and immune-histochemistry, and gut microbiota to disease resistance and stress tolerance, the immunological and hematological response, body composition, malformations, and the survival rate [122,123,124,125,126]. However, there is a gap in the literature on the use of *S. boulardii* and its effects on the intestinal immune system of fish. These findings indicate that yeast products are thought to be more effective as immunostimulants than immunizations and antibiotics to reduce fish mortality.

The results of the literature review demonstrate that the evaluated yeast products effectively functioned as prebiotics and probiotics. The separation of the two previous chapters was solely for the purpose of providing an overview of the discussed questions. However, in experimental practice, these are inseparable, given the methodology that is typically applied

## 6. Digestibility of Yeast in Fish and Crustaceans

Digestibility is an important characteristic of yeast that makes it an economically competitive protein source for aquaculture, comparable to conventional sources in various fish species’ diets [15,18]. Yeast cell walls represent 26–32% of the cell dry weight and contain varying proportions of mannan-oligosaccharides, *β*-glucan, chitin, and nucleic acids, depending on the species. In addition, the cell wall fraction contains bioactive substances like as β-glucan and mannan oligosaccharides, which promote protein solubility and digestibility during in vitro digestion [127]. Increasing replacement of fishmeal with *S. cerevisiae* in the latter study also resulted in slightly decreasing apparent digestibility of dry matter. Baker’s yeast is a type of probiotic that can boost immunity, supply an enzyme for food digestion, and improve feed intake [108]. In a study, the effect of adding 10% or 20% torula yeasts to a fishmeal-free diet on the digestibility and growth of juvenile rainbow trout was evaluated. The results of this study showed that when fed a diet devoid of fishmeal, torula yeast (*C. utilis*) may help rainbow trout grow more robustly [6]. Brewer’s yeast significantly decreased digestibility coefficients of dry matter, protein, and energy in sea bass juveniles when fishmeal was partially replaced with yeast [15]. Arctic char had lower energy and amino acid digestibility than extracted *S. cerevisiae*, but there was no significant difference in Eurasian perch (*Perca fluviatilis*) [41]. This demonstrates variances in digestive efficiency among species, probably related to varying gastrointestinal enzyme activity [128]. In studies with Atlantic salmon, the replacement of 40% of low temperature (LT) fishmeal with *C. utilis* or *K. marxianus* yeast had no significant effect on the digestibility of crude protein and energy, whereas the substitution with *S. cerevisiae* significantly reduced protein and energy digestibility [36]. Protein digestibility in Atlantic salmon was further enhanced by the improved solubility of proteins and β-glucans resulting from distinct downstream processing techniques of the yeast *S. cerevisiae* [127]. The digestibilities of individual amino acids were also unaffected by the dietary inclusion of *C. utilis* and *K. marxianus*, except for a reduced digestibility of methionine and cysteine in the *K. marxianus* diet. Conversely, the diet containing *S. cerevisiae* revealed significantly lower amino acid digestibility than the fishmeal control except for glycine and cysteine [36]. The latter study [15] indicated considerable differences in digestibility among different whole-cell yeast products. The enzymatic hydrolysis of cell wall rupture can improve the digestibility of yeast nutrients [109]. Moreover, by removing the cell wall material, which is a representation of the water-soluble cell contents, yeast can be extracted from lignocellulosic biomass and used as aquaculture feed. Since cell walls are removed and there is a greater amount of water-soluble low molecular weight proteins in the diet, adding more yeast extract in place of fishmeal in shrimp (*Litopenaeus vannamei*) diets increased the apparent digestibility of protein [129]. The extrusion of feed may cause partial disruption of yeast cell walls and potentially increase protein and amino acid digestibility [130]. Enhanced growth performance and health in fish fed on yeast might also be attributed to the improved digestive enzyme activity [111] and by supplying digestive enzymes that aid in the digestion of complex carbohydrates, [131] or by enhancing digestive capacity by improving gastrointestinal morphology and thereby increasing the absorptive surface [36,109,111].

## 7. Use of Yeast in Live Aquafeed Culture

The significant advancements in technology for larval rearing, along with the global proliferation of hatcheries, necessitate the use of appropriate rotifer culture techniques as a vital source of larval nourishment. Rotifers (*Brachionus* sp.) are supplied as a living capsule, providing the necessary nutrients for the proper growth and production of cultivated fish larvae [132]. In addition, rotifers are the most important live-feeding species utilized in the rearing of fish larvae because of their size, nutritional content, and behavior [18,133]. Furthermore, it has been proved by many researchers that a medium including yeast, starch, and albumen is suitable for the rotifer culture. In addition, by using yeasts in aquaculture, copepods, artemia, and rotifers can be produced along with live aquafeed [18]. Moreover, *Artemia nauplii* is now one of the most dependable live feeds for fish and is frequently utilized to nourish young aquatic species raised in aquaculture [134]. However, the amount of yeast used in rotifer culture varies greatly due to insufficient information on feeding rate, frequency, and composition range [135]. Through the use of a somewhat diet-dependent procedure with variable yeast amounts, the ideal feeding rate and frequency for *Brachionus plicatilis* was found [136]. The results showed that the feeding rate of the rotifers is sufficient at an average of 0.3 g of barker yeast per million rotifers. Additionally, increasing the feeding frequency can lead to a higher population growth rate and egg-bearing ratio, with an increase from twice to three times [136]. According to a study, yeast works better for Rotifer *Brachionus calyciflorus* cultures when combined with fresh *Chlorella* compared to powdered *Chlorella*. It is advised that better raising conditions will enable good rotifer development when the food contains both fresh *Chlorella* and yeast [133]. Furthermore, research has demonstrated that yeast increases the density of copepods in culture, suggesting that it could one day be used as a supplement to feed for additional copepod species [137]. According to other studies, the maximum relative population density of copepods has been reached when compared to marine microalgae when copepods are fed commercial *S. cerevisiae* baker’s yeast with a soybean component [138]. Therefore, in the cultivation of live food, yeast is recommended as a supplement or protein alternative.

The application of live feed is a standard part of the production of some fish species, which is important from both a commercial and a nature conservation standpoint. The results of the experiments outlined above clearly demonstrate that yeast products are an excellent source of nutrition for rotifers and microcrustaceans used as live food.

## 8. Conclusions and Aspects of Future Research

In conclusion, the use of yeast as a primary protein-rich feed additive, supplement, and probiotic has the potential to provide the necessary solutions to meet current and future market demands and address issues associated with the production of several valuable species for the future of the aquaculture sector. Furthermore, the current investigation has demonstrated that the addition of yeast to the diets of several fish species can enhance their growth, immunity, and efficiency in utilizing nutrients. Our review of the literature indicates that the addition of yeast to fish feed, either directly or as a supplement, can facilitate growth, improve digestive efficiency, and bolster immune systems. Furthermore, the use of yeast as a probiotic can enhance immune systems and improve water quality, thereby increasing yield. However, to maximize growth, survival, and health outcomes, it is recommended to use probiotics in exploitable species. Furthermore, yeast is regarded as a viable option for live aquafeed and aquaculture. It is not recommended to substitute all other protein sources in aquafeed with yeast proteins, as this could have adverse effects on certain fish species and pose potential health risks to consumers.

However, the efficacy of yeast as a primary protein source in fish diets is still under investigation, and further research is necessary. Yeast products have been shown to have a protein digestibility that is similar to that of conventional protein sources when incorporated into the diets of different fish species. However, there is a lack of research comparing the digestibility of different yeasts in the same experiment. To compete with fishmeal in the aquaculture sector and become a reasonably priced alternative for fish farmers, large-scale investment is required for yeast production. This would also help to reduce their dependency on fishmeal. While yeast can be used as a protein source in aquafeed, there is currently limited knowledge regarding its safety. To prioritize the development of potential ways that address fish and environmental safety and human health, more scientific research on the use of yeast in the aquaculture industry is required. Future research should focus on understanding the specific nutritional needs of cultured aquatic species at different stages and developing affordable dry feeds that the industry can use.

## Figures and Tables

**Table 1:** Chemical composition of *Saccharomyces cerevisiae* as yeast meal and fishmeal.

Composition	*Saccharomyces cerevisiae* from Beer Fermentation	Menhaden Fishmeal
Dry matter%	93	91.2–92
ME (kcal/kg)	1990	3370
Crude protein%	44.4	59–68.5
Crude fat%	1	9.1–10.4
Crude fiber%	2.7	0.9
Ca%	0.12	4.87–5.34
P%	1.4	2.93–3.05

Source: [28,29,30].

**Table 2:** Yeast species used in aquaculture as a primary protein source to enhance growth performance, disease resistance, and immune system of different fish species.

Product Name	Fish Species	Inclusion Levels	Effects	Source
Brewer’s yeast	Largemouth bass *(Micropterus salmoides)*	20, 40, 60 g/kg	The supplementation of *S. cerevisiae* at 10 g kg^−1^ did not significantly reduce fishmeal in the diet.	[50]
Dietary yeast polysaccharides	Channel catfish *(Ictalurus punctatus)*	20 g/kg	The study found that fish fed experimental diets for 12 weeks significantly improved growth performance compared to the control.	[50]
Yeast, *Saccharomyces* *cerevisiae* (SC)	Gilthead sea bream *(Sparus aurata)*	1, 2, and 4 g/kg	The study showed that at a dose of 4 g/kg, a meal high in SC dramatically improved growth performance, intestinal morphology, redox homeostasis, and immunological response.	[45]
Protein-rich yeast fraction	Rainbow trout *(Oncorhynchus mykiss)*	0, 5, 10, or 15%	The use of yeasts in 60% of fishmeal protein can cause haemolytic anemia in rainbow trout, potentially restricting their inclusion in farmed fish diets.	[13]
Dietary yeast hydrolysate	Nile tilapia *(Oreochromis niloticus)*	1 and 3%	1% yeast hydrolysate supplementation enhances growth performance, feed utilization, and antioxidant status.	[51]
Baker’s yeast	Freshwater catfish gulsa tengra *(Mystus cavasius)*	0.5, 1, and 1.5 g/kg	Yeast supplement is a potentially effective growth promoter that could replace antibiotics in the treatment of *M. cavasius* infections.	[42]
Dietary hydrolyzed yeast	Pacific white shrimp *(Litopenaeus vannamei)*	1%	The study found no significant effect on growth performance or body composition, but it did affect intestinal health, immunological responses, and ammonia resistance.	[52]
Autolyzed dried yeast	Gilthead sea bream *(Sparus aurata)*	0 and 5%	Following a 92-day feeding trial, there were no discernible variations between the dietary groups in terms of fish growth rate, mortality, or feed efficiency.	[53]
Dietary yeast hydrolysate	Largemouth bass *(Micropterus salmoides)*	0, 1.5, 3.0, 4.5%	Dietary yeast hydrolysate has been found to enhance the antioxidant capacity and immune response of largemouth bass without any adverse growth effects.	[54]
Yeast (*S. cerevisiae*)	Sea cucumber, *(Apostichopus japonicus)*	5%	The study aimed to enhance the growth, digestive enzyme activity, nutritional value, and immune system of sea cucumber juveniles.	[55]
Yeast, *Saccharomyces* *cerevisiae*, extract	Juvenile pikeperch *(Sander lucioperca L.)*	2, 4, and 6%	The study found that pikeperch growth is stimulated by the lowest analyzed dose of yeast, which is 2% yeast extract.	[56]
Yeast culture (YC)	Gibel carp *(Carassius* *auratus gibelio CAS Ⅲ)*	0, 20, 40, and 60%	The study suggests that yeast culture could be a suitable fishmeal alternative for Gibel carp diets, with a dietary inclusion of 4 g of yeast culture per 100 g diet.	[57]
Yeast hydrolysate (YH)	Juvenile Jian carp *(Cyprinus carpio var.* Jian*)*	1, 3, 5, and 7%	The study found that 3% YH was the most effective substitution for fishmeal (FM) in enhancing innate immunity and growth performance.	[58]
Dried yeast	Red drum *(Sciaenops* *ocellatus)*	20, 30, 40, 50%	Without impairing the red drum’s functionality, the material may replace between 30 and 50% of the protein supplied by fishmeal.	[49]
Brewer’s yeast (*Saccharomyces cerevisiae*)	Thai Panga *(Pangasianodon hypophthalmus ×* *Pangasius bocourti)*	30, 45, 60, 75%	By substituting brewer’s yeast for 45% of the fishmeal, one can improve the immune response and growth performance of the Thai Panga.	[48]
Brewer’s yeast	Goldfish *(Carassius* *auratus)*	0, 15, 25, 35, and 45%	Fish fed a diet with yeast replacing 35% of their meal showed better weight gain, SGR, FCR, and protein efficiency ratio compared to other diets.	[59]
Dietary yeast cell wall (YCW)	Japanese seabass *(Lateolabrax japonicus)*	0, 250, 500, 1000, 2000, and 20,000 mg/kg	The study found that the optimal dose of YCW is 2000 mg/kg, with a 10 × safety margin.	[60]
Yeast (*Saccharomyces cerevisiae)*	Juvenile channel catfish *(Ictalurus punctatus)*	25, 50, 75, 100, and 125 g/kg	The study found that adding up to 100 g kg^−1^ of dried yeast without affecting growth performance is possible.	[61]
Baker yeast, *Saccharomyces cerevisiae* (SC)	Nile tilapia *(Oreochromis niloticus)* fingerlings	1 and 2 g/100 g	Nile tilapia fingerlings fed a yeast mixture for 119 days showed improved growth performance, feed efficiency, and hematological indices.	[62]
Ethanol yeast (EY)	Sunshine bass (female white bass *Morone* *chrysops ×* male striped bass *M. saxatilis)*	0, 7.5, 15%	Reductions in carcass protein and increase in carcass lipid was the results of replacing fishmeal entirely with EY. It also caused changes in whole-body composition.	[63]
Brewer’s yeast	Sea bass *(Dicentrachus labrax)*	0, 10, 20, 30, or 50%	Brewer’s yeast can replace 50% of fishmeal protein without negative effects on fish performance and improve feed efficiency by up to 30% in the diet.	[64]
Brewer’s yeast (*Saccharomyces cerevisiae*)	Beluga sturgeon *(Huso huso)* juveniles	1 and 2%	Brewer’s yeast can enhance growth performance and modify intestinal microbiota in beluga sturgeon without negatively impacting basic hematological parameters.	[65]
Yeast-based, certified organic protein source	Cobia *(Rachycentron canadum)*	25, 50, 75, and 100%	The data indicates that at least 25% of dietary protein can be provided by yeast-based protein in diets for cobia, despite negative effects on production characteristics.	[66]
Brewer’s yeast (*Saccharomyces cerevisiae*)	Hybrid striped bass *(Morone chrysops ×* *M. saxatilis)*	1, 2, and 4%	The growth performance, feed efficiency, and infection resistance of hybrid striped bass were found to be greatly improved by brewer’s yeast.	[67]

**Table 3:** Yeast products used in aquaculture as supplementary protein sources to enhance growth performance, disease resistance, and the immune system of several species.

Product Name	Fish Species	Inclusion Levels	Effects/Outcome	Source
Torula Yeast (*C. utilis*)	Rainbow trout *(Oncorhynchus mykiss)*	10 and 20%	The findings of this study suggested that torula yeast (*C. utilis*) may promote more robust rainbow trout growth when given a diet free of fishmeal.	[6]
Selenium yeast	Golden pompano *(Trachinotus ovatus)*	1 g/kg	The study suggests that golden pompano can be reduced in dietary fishmeal to 1 g/kg Selenium-yeast.	[73]
*Saccharomyces cerevisiae-* fermented poultry byproduct meal (pbm)	Common carp *(Cyprinus carpio)*	0, 5, 10, 15, 20%	The inclusion of 15–20% yeast and fermented poultry byproduct meal in the common carp’s diet has been proven to enhance digestive enzyme activity, immunological function, and growth.	[74]
Dietary inclusion of fermented poultry byproduct meal (FPBM)	Nile tilapia *(Oreochromis niloticus)*	10, 20, 30, and 40%	To improve tilapia health and growth, 11.17–25.14% of FPBM can be added to their diets in an efficient manner.	[22]
Yeast cell wall	Juvenile Persian sturgeon *(Acipenser persicus)*	0.5 and 1%	The growth metrics and feeding performance are not significantly affected by the administration of 0.5 and 1% Immunowall.	[76]
Selenium yeast (Se)	Meagre *(Argyrosomus regius)* fingerlings	0.77, 1.51, 2.97, and 3.98 mg /kg	To improve the development performance, feed utilization, liver and kidney histology, and economic benefits of meagre juveniles, a meal intake of 3.98 mg Se kg−1 (3 mg Se-yeast) is advised.	[77]
Yeast-fermented sunflower meal (YFSFM)	Nile tilapia *(Oreochromis niloticus)*	0, 25, 50, and 75%	The varying levels of YFSFM had no significant effect on the fish’s dry matter, lipid, crude protein, or ash content.	[78]
Selenium yeast	Siberian Sturgeon *Acipenser baerii*	5, 10, 15 g/kg	A food supplement of Sel-Plex at levels ≤15 g kg−1 increased the growth performance of Siberian sturgeons even though the hematological indicators improved.	[79]
Substituting soybean meal with yeast (*Sacharomyces cerevisae*) meal	African catfish *(Clarias gariepinus)*	0, 10, 20, 30, 40, 50, and 100	The diet with a 50% yeast inclusion was deemed optimal due to its enhanced nutritional status, improved blood parameters, and enhanced fish health.	[80]
Grain distillers dried yeast (GDDY)	Juvenile rainbow trout *(Oncorhynchus mykiss)*	0, 25, 37.5, 50, 62.5, 75, 87.5, and 100%	Rainbow trout development and feed conversion were highly affected by high GDDY incorporation rates, but feed intake was not affected.	[81]
Yeast-fermented canola meal	Nile tilapia *(Oreochromis niloticus)*	0, 25, 50, 75, and 100%	The study found that replacing fish with 75 and 100% levels significantly reduced their protein efficiency ratio and nutrient digestibility compared to lower levels.	[82]
Yeast, Metschnikowia sp. in combination with Rhodotorula	Juvenile of sea cucumber *(Apostichopus japonicus)*	104, 105, and 106 CFU g−1	Boost the immune system, boost growth, increase the synthesis of digestive enzymes, and provide nourishment.	[83]
Yeast-fermented canola meal	Asian sea bass *(Lates calcarifer)*	25, 50, 75, 100%	The study revealed that yeast-fermented canola meal can replace 50% of fishmeal in the Asian sea bass diet without affecting growth.	[84]
Fry Fed Organic Diets Containing Yeast Extract (YE)	Nile tilapia *(Oreochromis niloticus)*	0, 15, 30, and 45%	Fry fed a control diet with 20% FM and a diet with 45% YE/36%SBM with amino acid supplementation showed no significant differences in final weight, weight gain, and specific growth rate.	[85]
Ethanol yeast	Sunshine bass *(Morone chrysops ×* *M. saxatilis)*	0, 7.5, 15, and 22%	According to the study, sunshine bass should be fed ethanol yeast-based diets with an FM level of between 7.5% and 15%.	[63]
Dietary supplementation with brewer’s yeast	Red drum *(Sciaenops ocellatus)*	10 g/kg	The study suggests that a ten-dose dose of several prebiotics is sufficient to enhance the feed efficiency and disease resistance of red drums.	[86]
Yeast protein *Saccharomyces cerevisiae* supplemented with biogenic L-carintine	Nile tilapia *(Oreochromis niloticus)* *fingerlings*	25, 50, 75, and 100%	Tilapia fed a diet containing 7.14 and 10.71% yeast, supplemented with 100 mg/100 g diet, showed optimal growth performance, feed and protein utilization.	[87]
Yeast extract powder (YEP)	Rohu *(Labeo rohita)* fingerlings	0.1, 0.2, 0.3, 0.4, and 0.5%	Up to a 0.2% margin, the fish fed diets enriched with YEP grew more rapidly than the control group.	[88]
Brewer’s grains with yeast (BGY)	Australian red claw crayfish *(Cherax quadricarinatus)*	10, 20, and 30%	The study suggests that soybean meal and BGY can be completely replaced with fish and shrimp meal in the diets of juvenile red claw crayfish.	[89]

**Table 4:** Yeast products used in aquaculture as probiotics to enhance growth performance, disease resistance, and immune system of different species.

Fish Species	Inclusion Levels	Effects	Source
Pacific white shrimp (*Litopenaeus vannamei*)	10 g/kg	According to the study, adding yeast byproduct additions to sugar byproducts may enhance development, immunity, histological changes, and resistance to the AHPND-causing *V. parahaemolyticus*.	[94]
Siberian Sturgeon *(Acipenser baerii)*	50% Lactic acid + 50% *Saccharomyces boulardii*	Growth indices, survival, and well-being of *Acipenser baerii* fish were dramatically improved when probiotics, such as lactic acid bacteria and yeasts, were introduced to their meals.	[92]
Persian sturgeon *(Acipenser persicus)*	5 g	This study contributes to the growing literature on the effects of probiotics on the growth, body composition, digestive enzymes, and intestinal morphology of *A. persicus* fingerlings.	[99]
Gilthead sea bream *(Sparus aurata)*	0.55 or 1.1%	The study found that yeast, when consumed at a 0.55 or 1% basal diet, exhibited immunostimulant activity in gilthead seabream.	[106]
Rainbow trout, *(Oncorhynchus mykiss fry)*	1, 5, and 10%	Adding yeast to rainbow trout fry diets during early life stages is suitable, with a 5% concentration likely enhancing growth performance and feed efficiency ratio.	[107]

## Data Availability

No new data were created or analyzed in this study. Data sharing is not applicable to this article.
